# PSN-PC: A Novel Antimicrobial and Anti-Biofilm Peptide from the Skin Secretion of *Phyllomedusa-camba* with Cytotoxicity on Human Lung Cancer Cell

**DOI:** 10.3390/molecules22111896

**Published:** 2017-11-07

**Authors:** Xianhui Wu, Jinhuo Pan, Yue Wu, Xinping Xi, Chengbang Ma, Lei Wang, Mei Zhou, Tianbao Chen

**Affiliations:** 1 School of Pharmacy, Nanjing University of Chinese Medicine, Nanjing 210000, China; wxhui2013@163.com; 2 Natural Drug Discovery Group, School of Pharmacy, Queen’s University, Belfast BT9 7BL, Northern Ireland, UK; ywu16@qub.ac.uk (Y.W.); x.xi@qub.ac.uk (X.X.); c.ma@qub.ac.uk (C.M.); l.wang@qub.ac.uk (L.W.); m.zhou@qub.ac.uk (M.Z.); t.chen@qub.ac.uk (T.C.)

**Keywords:** antimicrobial peptide, phylloseptin, anti-biofilm activity, cancer cell cytotoxicity

## Abstract

Peptides derived from amphibian skin secretion are promising drug prototypes for combating widespread infection. In this study, a novel peptide belonging to the phylloseptin family of antimicrobial peptides was isolated from the skin secretion of the *Phyllomedusa camba*, namely phylloseptin-PC (PSN-PC). The biosynthetic precursor was obtained by molecular cloning and the mature peptide sequence was confirmed through tandem mass spectrometry (MS/MS) fragmentation sequencing in the skin secretion. The synthetic replicate exhibited a broad spectrum antimicrobial activity against *Staphylococcus aureus*, methicillin-resistant *Staphylococcus aureus,* *Escherichia coli*, *Pseudomonas aeruginosa*, *Candida albicans* at concentrations of 2, 2, 8, 32 and 2 µM, respectively. It also showed the capability of eliminating *S. aureus* biofilm with a minimal biofilm eradication concentration of 8 µM. The haemolysis of this peptide was not significant at low concentrations but had a considerable increase at high concentrations. Additionally, this peptide showed an anti-proliferation effect on the non-small cell lung cancer cell line (NCI-H157), with low cytotoxicity on the human microvascular endothelial cell line (HMEC-1). The discovery of the novel peptide may provide useful clues for new drug discoveries.

## 1. Introduction

Amphibian skin secretion consists of various bioactive peptides that play an essential role in amphibian survival. These peptides demonstrated multifunctional activities against Gram-negative and Gram-positive bacteria, fungi, enveloped viruses and even cancer cells [1]. Additionally, some other peptides are considered potentially to be immunomodulatory and anti-diabetic agents [2], and even contraceptives, as they can be cytotoxic to sperm [3]. These skin-derived antimicrobial peptides (AMPs) are especially predominant and demonstrate board spectrum antimicrobial activity, which make them potential drug candidates in the treatment of bacterial infections. These AMPs are able to efficiently kill antibiotic-resistant bacteria and have little chance for inducing serious drug resistance [4]. Some AMPs have been reported to be capable of inhibiting biofilm formation as well as eradicating mature biofilm [5], which makes bacteria 1,000-fold more resistant to conventional antimicrobial agents than their planktonic counterparts.

Among all the AMPs, α-helical antimicrobial peptides, such as magainins [6] and dermaseptins [7], have been extensively studied regarding their biosynthesis, antimicrobial activity, mechanism of action, three-dimensional structures, and further applications. However, the phylloseptins have not attracted much attention. Several phylloseptins were first identified in 2005, showing anti-bacterial and anti-protozoan peptide activities, from the skin secretion of the Brazilian tree-frogs, *Phyllomedusa hypochondrialis* and *Phyllomedusa oreades* [8]. Besides, Yasser et al. found a phylloseptin from the skin of the frog *Hylomantis lemur* induced insulin release from the rat BRIN-BD11 clonal β cell line, providing a promising alternative for treating Type 2 diabetes [9].

In the present study, the isolation of a novel phylloseptin precursor from the *Phyllomedusa camba* skin secretion, which has been rarely studied so far, was conducted by ”shotgun” molecular cloning. The primary structure of the peptide was confirmed by tandem mass spectrometry (MS/MS) fragmentation and named phylloseptin-PC (PSN-PC). The replicate was then chemically synthesised and purified for downstream study. Subsequently, experiments were designed to evaluate the effects of PSN-PC on microorganisms, cancer cell lines and its toxicity to horse erythrocytes and normal human cells of the human microvascular endothelial cell line (HMEC-1). Furthermore, the secondary structure of this novel peptide was predicted and time-killing curves along with bacterial cell membrane permeability assays were performed to explore its mechanism of action. 

## 2. Results

### 2.1. Molecular Cloning of a Novel AMP Precursor-Encoding cDNA and Bioinformatic Analyses

**Figure 1:**
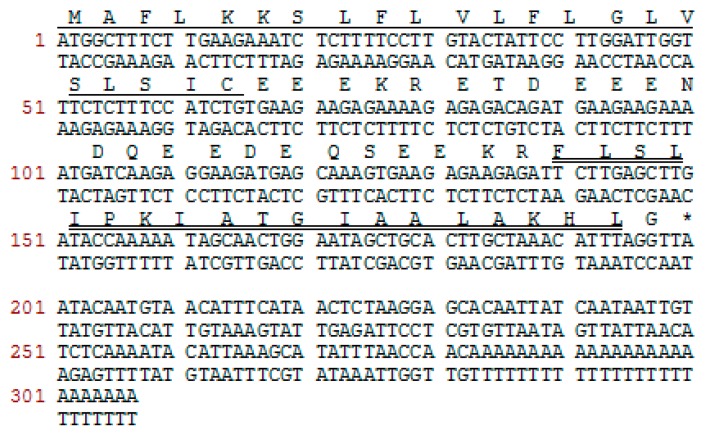
Nucleotide and translated open-reading frame amino acid sequence of biosynthetic precursor cDNA encoding the novel mature peptide. The putative signal peptide is single-underlined, the mature peptide is double-underlined, and the stop codon is indicated by an asterisk.

**Figure 2:**
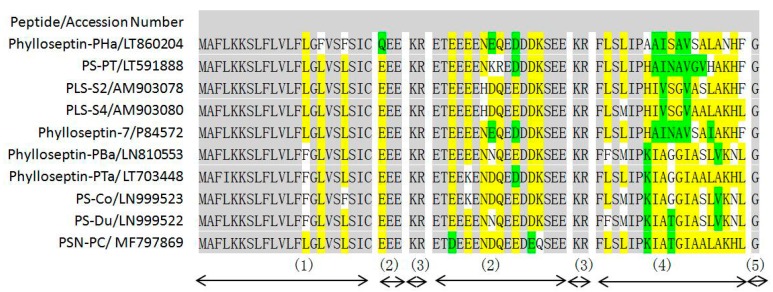
Multiple alignments of the cloned cDNA-deduced amino acid sequence of phylloseptins with antimicrobial activities. Grey shading indicates identical amino acid residues, yellow shading indicates consensus amino acid residues, and green shading indicates similar amino acid residues. (1): putative signal peptide; (2): acidic spacer peptide region; (3): dibasic propeptide convertase processing site; (4): mature peptide; (5): glycine residue amide donor.

A full-length cDNA encoding the biosynthetic precursor of PSN-PC was consistently and successfully cloned from the skin secretion library (Figure 1). The alignment of phylloseptins shows that the members share a highly-conserved amino acid sequence in the phylloseptin family (Figure 2). There were several typical characteristics in the translated open reading frame: (1) a highly-conserved putative signal peptide region of 22 amino acid residues, which is homologous to each other; (2) acidic spacer peptide region consisting of Glu, Asp and other hydrophilic amino acids; (3) a classical propeptide convertase processing site (-KR-); (4) a mature active peptide encoding domain that contained 19 amino acid residues; and (5) the C-terminal glycine residue acted as an amide donor. The significant variations occurred in the mature peptide domain were at the position 7, 9, 10, 13, 14 and 15. The nucleotide sequence of this PSN-PC precursor was deposited in the Genbank Nucleotide Sequence Database under the accession code, MF797869.

### 2.2. Fractionation of Skin Secretion, Identification and Structural Characterisation of PSN-PC

**Figure 3:**
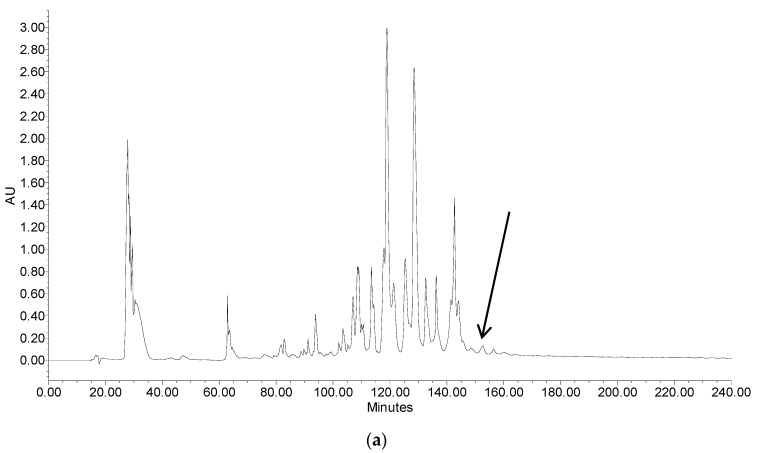

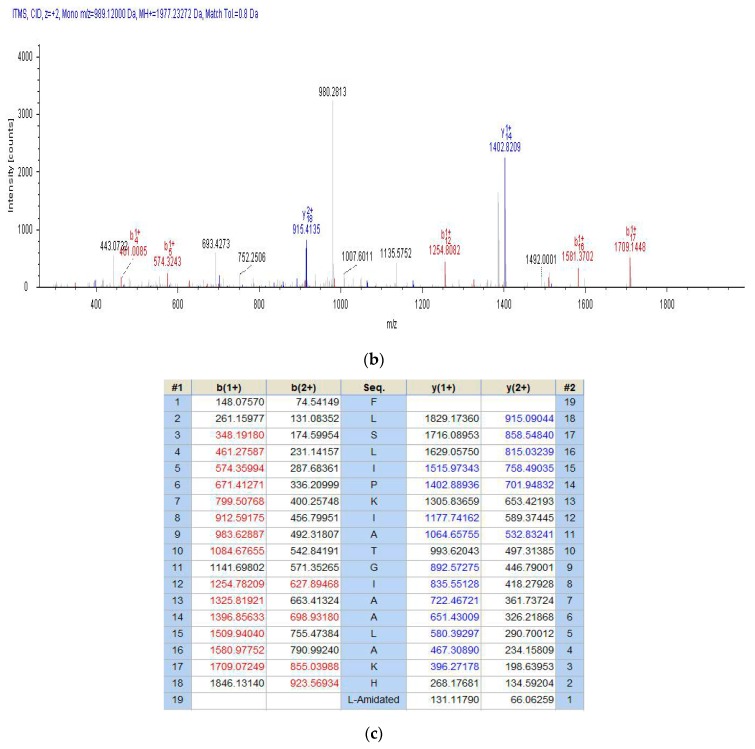
(**a**) Reverse phase high performance liquid chromatography (HPLC) chromatogram of skin secretion of *Phyllomedusa camba* monitored at 214 nm. The arrow indicated the retention time of PSN-PC; (**b**) Tandem mass (MS/MS) fragmentation spectrum of PSN-PC; (**c**) Predicted singly-charged b ions and y ions arising from MS/MS fragmentation. The observed b- and y-ions were indicated in blue and red typefaces.

The fractions of skin secretion resolved by reverse-phase high performance liquid chromatography (RP-HPLC) are shown in Figure 3a, with an arrow indicating the retention time/elution position of the peptide with the predicted peptide mass. The fraction that yielded the predicted peptide mass was further analysed by MS/MS fragmentation (Figure 3b,c). The ion 980.28 *m*/*z* was considered as a NH_3_ loss from the parent ion, which also indicated the C-terminal amindation.

### 2.3. Conformational Study

**Figure 4:**
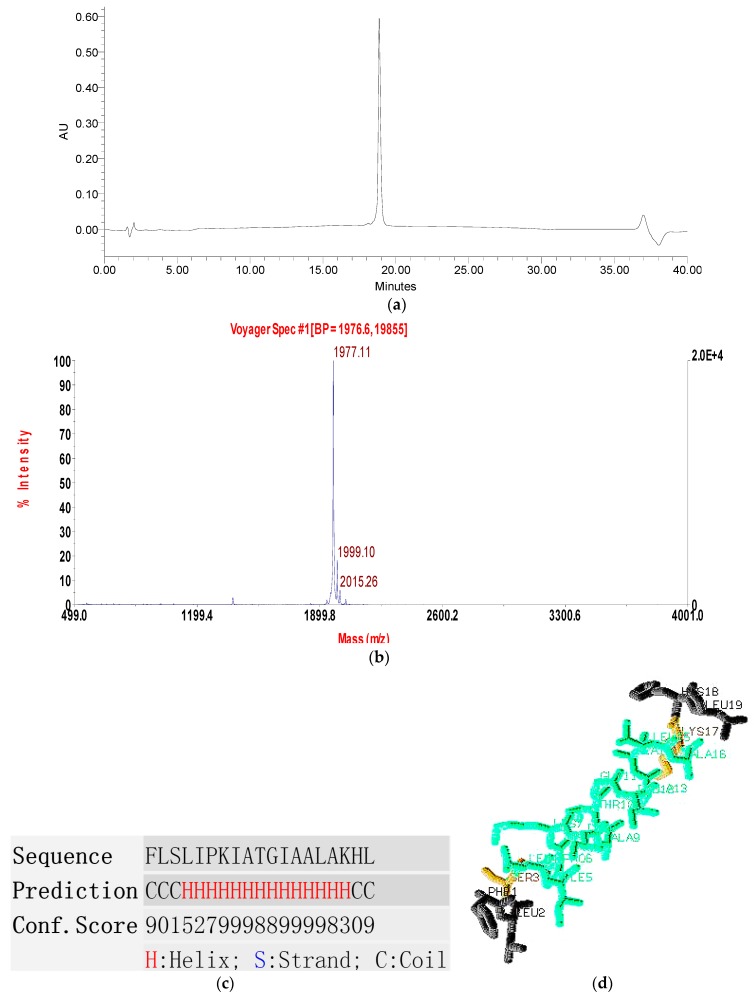

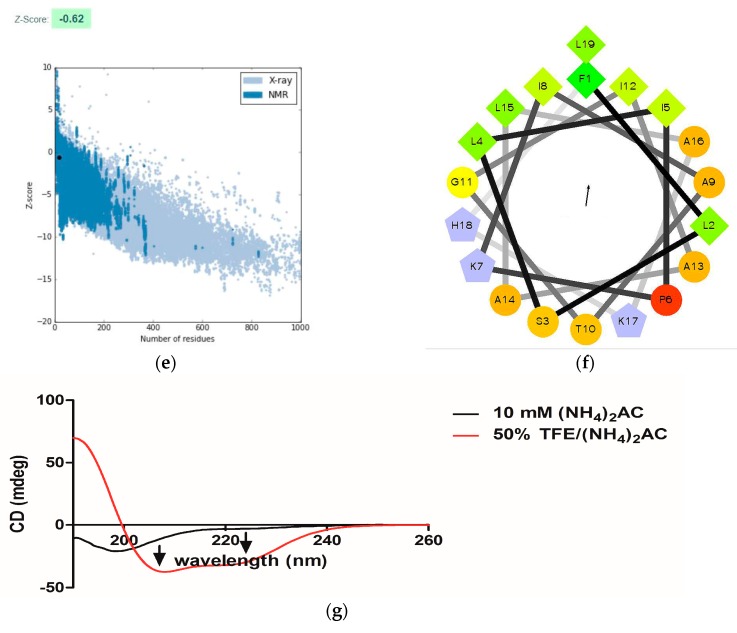
(**a**) The RP-HPLC chromatogram of the purified synthetic phylloseptin-PC (PSN-PC); (**b**) MALDI-TOF mass spectrum of synthetic PSN-PC; (**c**) Predicted secondary structure of PSN-PC using I-TASSER; (**d**) Predicted 3D model of PSN-PC using I-TASSER; (**e**) Z-score plot using ProSA-web; (**f**) Helical wheel plot of PSN-PC; (**g**) Circular dichroism (CD) spectra recorded for PSN-PC (100 μM) in 10 mM ammonium acetate buffer and 50% TFE ammonium acetate buffer.

The purified product of solid-phase peptide synthesis was successfully obtained by RP-HPLC and MALDI-TOF MS with a high degree of purity (Figure 4a,b). The observed molecular weight of PSN-PC was 1976.11Da (Figure 4b) which was consistent with that of the natural peptide. This peptide contained a large proportion of α-helical domain with a series of high scores representing a more confident prediction of secondary structure (Figure 4c). Similarily, a three-dimensional simulation of the synthetic peptide exhibited the structural feature of coil-helix-coil (Figure 4d). Z-score of PSN-PC was within the range of scores typically found for native proteins of similar size, indicating that the overall model quality is reliable (Figure 4e). Moreover, the helical wheel projection predicted that the peptide had an obvious propensity for the α-helix formation which was typical in most AMPs (Figure 4f). An amphipathic structure was observed with the hydrophobic residues (L4, L15, I8, F1, L19, I12, I5, A16, A9) and cationically hydrophilic residues (H18, K7, K17) partitioning on opposites of the molecule. Finally, the circular dichroism (CD) results of PSN-PC exhibited an unordered structure in 10 mM ammonium acetate buffer but typically α-helical bands at around 208 nm and 220 nm in 50% TFE ammonium acetate buffer (Figure 4g). These typical characteristics suggest that PSN-PC adopt a well-defined α-helical structure when in contact with a hydrophobic environment.

### 2.4. Bioactivity Assays of PSN-PC

**Figure 5:**
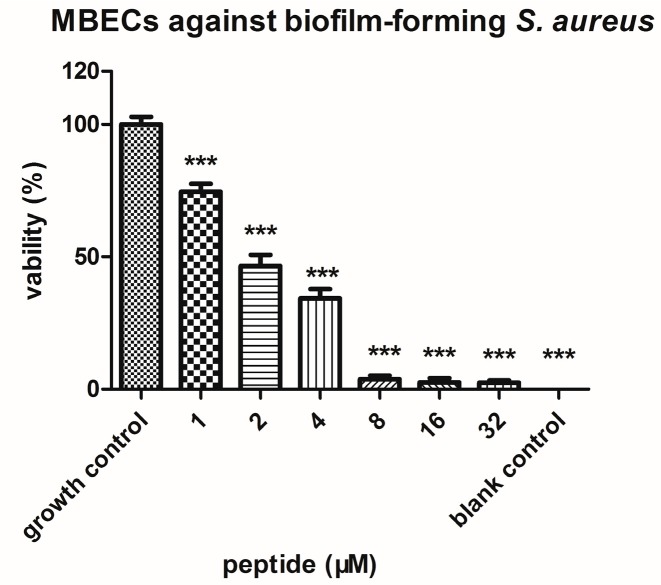
The MBEC (minimal biofilm eradication concentration) of PSN-PC against *S. aureus* biofilm. The results were analysed by one-way ANOVA, followed by the Newman-Keuls test, and showed differences between growth control and all concentrations of PSN-PC (***) *p* < 0.001.

The peptide tested possessed significant bioactivity against Gram-positive bacteria *Staphylococcus aureus*, methicillin-resistant *Staphylococcus aureus* (MRSA) and the yeast Candida albicans at the same minimum inhibitory concentration (MIC) of 2 µM (Table 1). This peptide was also potent against the Gram-negative bacteria *Escherichia coli* and *Pseudomonas aeruginosa* (MIC of 8 and 32 µM, respectively). The MIC values of the phylloseptin were aligned in Figure 2 and their corresponding physicochemical parameters listed in Table 2 [8,10,11,12,13,14,15]. PSN-PC demonstrated the strongest antibacterial activity against the growth of *S. aureus* and *C. albicans*. It exhibited ~2-fold more potent antimicrobial activity than Phylloseptin-PBa, Phylloseptin-PTa, PS-Co and PS-Du against *S. aureus*. Also, PSN-PC showed slightly weaker inhibitory effects than Phylloseptin-7 against *E. coli*. Besides, it eradicated mature *S. aureus* biofilm at a minimal biofilm eradication concentration (MBEC) of 8 µM (Figure 5). 

**Figure 6:**
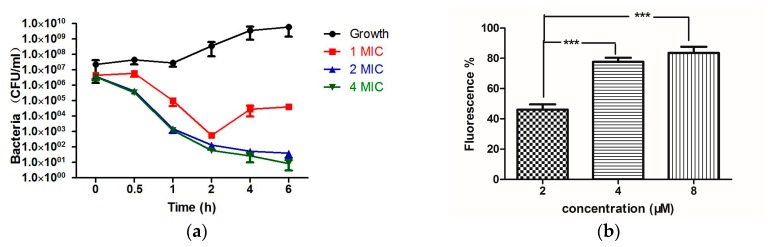
(**a**) Time-killing curves for PSN-PC on *S. aureus*. The antimicrobial peptide was added at time 0 h; (**b**) Cell-membrane permeability effects of *S. aureus* incubated with the peptide. The results were analysed by one-way ANOVA, followed by the Newman-Keuls test, and showed differences between 2, 4 and 8 µM (**b**) (***) *p* < 0.001.

The number of bacteria started to decrease in the first 30 min with the presence of 2 and 4 × MIC of PSN-PC, while the population remained constant at 1 × MIC (Figure 6a). During 0.5–2 h, all three PSN-PC concentrations were able to kill bacteria at similar rates, but 2 and 4 × MIC killed more bacteria, leading to 100 CFU/mL at 2 h. After that period, 2 and 4 × MIC of PSN-PC continued to kill bacteria until a state in which surviving cells could not be counted. While low doses of PSN-PC showed a recovery trend in the next 4 h. Further investigation of the cell-membrane permeabilisation of *S. aureus* after the 2 h incubation with corresponding concentrations of PSN-PC and bacteria cells were performed and the results showed that PSN-PC induced about 45% of cell-membrane permeabilisation at 2 µM on *S. aureus* (Figure 6b). Higher concentrations of PSN-PC led to ~2-fold increase of membrane permeabilisation.

**Figure 7:**
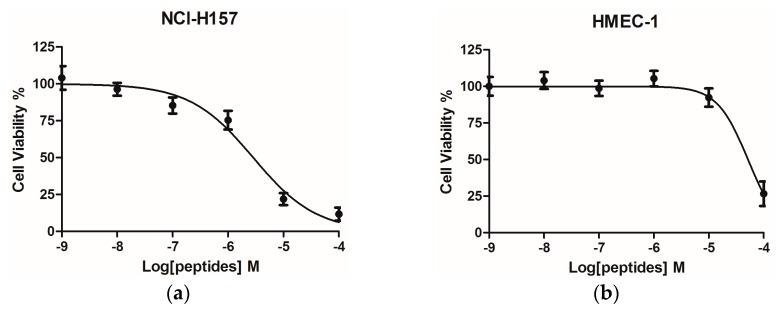
Dose-response curves of PSN-PC on the non-small cell lung cancer cell line NCl-H157 (**a**) and the human microvessel endothelial cell HMEC-1 (**b**) after 24 h incubation. IC_50_ of NCl-H157 and HMEC-1 were 2.85 and 51.83 µM, respectively.

**Figure 8:**
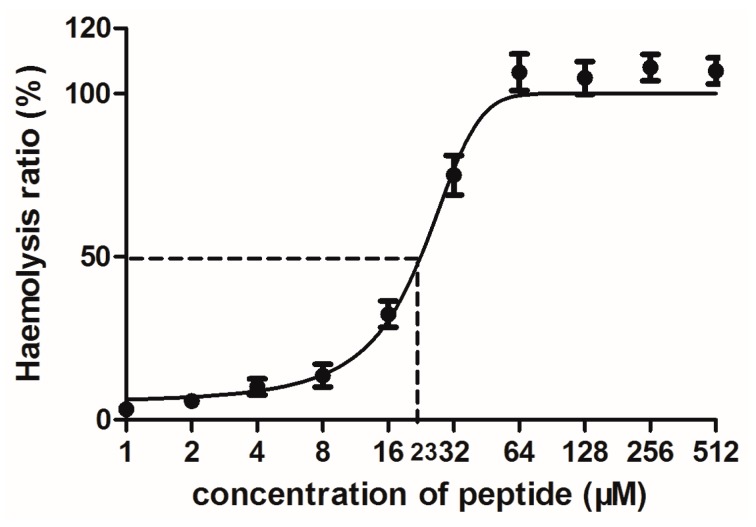
Haemolytic activity of PSN-PC. Percentage of haemolysis was calculated in comparison to the positive control using TritonX-100.

PSN-PC had a significant anti-proliferative effect on the non-small cell lung cancer cell line NCl-H157 with an IC_50_ of 2.85 µM (Figure 7a). Comparatively, the human microvessel endothelial cell line HMEC-1 was used to evaluate the inherent cytotoxicity of PSN-PC against normal human cells, which showed an IC_50_ of 51.83 µM (Figure 7b). PSN-PC had ~100% haemolysis at 64 µM, (Figure 8). The whole haemolytic process was divided into three parts, including a low degree of haemolysis at the initial stage (1–8 μM), a sharp rise in the middle stage (8–64 μM), and finally a constant period of complete haemolysis. The concentration of the test peptide which induced 50% haemolysis (HC_50_) was 23 μM.

## 3. Discussion

Peptides with biological activity from the skin secretions are considered to be an essential component of the innate immunity of amphibians. This ancient and diverse group of molecules provides protection for the host against various infections through rapid and broad-spectrum antimicrobial activities and immunomodulatory effects [16]. So far, 48 phylloseptins have been deposited in the Uniprot database and all were isolated from phyllomedusinae tree frogs (*P. sauvagii*, *P. bicolor*, *P. oreades* etc.) in South American countries, such as Brazil and Colombia [4,17]. In this study, PSN-PC was found from a virtually unstudied species of phyllomedusine frog in South America, *Phyllomedusa camba*, with homologies to other phylloseptins from *P. sauvagii*, *P. bicolor*, |*P. hypochondrialis* and *P. azurea* [18]. 

Phylloseptin peptides share common characteristics, such as a highly-conserved hexapeptide (FLSLIP-) in the N-terminal region (Figure 2) and C-terminal amidation, which has been shown to promote biological activity [19]. Lee et al. reported that a kink which was potentially introduced by proline was responsible for the helix distortion [20]. Importantly, Khara et al. found that backbone sequence comprising of (X_1_Y_1_Y_2_X_2_)_n_ (X_1_ and X_2_ are hydrophobic amino acids, Y_1_ and Y_2_ cationic amino acids, and n the number repeat units) exhibited broad-spectrum antimicrobial activities against drug-resistant and biofilm-associated infections [21]. In the phylloseptin family, the C-terminal tetrapeptide was consistent with the proposed motif. The aligned phylloseptin ending with a terminal Leu generally showed more potent antimicrobial activity than when ending with a Phe. The changes of antimicrobial effect may be related to the different amino acid compositions in the C-terminal tetrapeptide. Interestingly, the antimicrobial activity of phylloseptin could be affected by amino acid substitutions occurred at specific positions. For instance, Phylloseptin-PTa only had one amino acid difference with PSN-PC at position 10, from Gly to Thr, resulting in a 2-fold increase of antibacterial activity against *S. aureus* and *E. coli*. Additionally, the same change occurred between phylloseptin-PBa and PS-Du with antifungal activity reduced by half but with no changes in antibacterial activity. On the other hand, a Leu substituted by a Met at position 4, could generally result in relative higher antimicrobial efficacy. These common adaptations within peptide families not only shed light on phylogenetic information relevant to genetic mutations during natural selection, but also are helpful for future designs of antibiotic drugs.

Combining the results of time-killing curves and the cell membrane permeability assay (Figure 6), it is reasonable to think that the mechanism of action by 1 × MIC might be different from that at 2 × MIC (or 4 × MIC). It is presumed that at lower concentrations (1 × MIC), the peptide is unable to cover the entire lipid layer leading to the formation of transient, toroidal lipid-peptide pores only within a limited range, which agreed with a previously publication [22]. While at higher concentrations (2 or 4 × MIC), more complex antimicrobial mechanisms of mode may arise, such as a mixture of carpet mechanism and toroidal pore mechanism. Hallock et al. reported that MSI-78, an amphipathic α-helical AMP, resulted in the formation of a mixture of normal hexagonal phase and lamellar phase lipids at higher peptide concentrations [23].

Natural phylloseptins normally possess positive charges ranging from 0 to +2 (Table 2). Within a certain number of positively charged residues (usually 5–6), the increasing of net charge made these peptides more active for antimicrobial activity. Beyond the charge magnitude, the role of positive charges of a particular peptide can be limited [24]. PS-PT was an exception because its hydrophobicity (0.686) was lower than others (>0.7). Chen et al. claimed that there was an optimal hydrophobicity window in which high antimicrobial activity could be obtained. Decreased or increased hydrophobicity beyond this window resulted in a sharp decrease in antimicrobial efficacy [25]. Meanwhile, higher hydrophobicity was correlated with stronger haemolytic activity. This can be explained by the strong peptide self-association that hinders a peptide from passing through the cell wall in prokaryotic cells without affecting the access to eukaryotic membranes [26]. The peptide self-association might be an important reason for the significant haemolysis of PSN-PC (100% haemolysis at 64 µM). Park et al. synthesized an N-terminal random coil deleted A3-NT from an amphipathic AMP HP-A3, and the shorter peptide showed increased antibacterial and antifungal activity [27]. Physicochemical parameters, such as length, sequence, and hydrophobicity, are intimately correlated and replacing a single factor induces changes to the others.

Compared to published phylloseptins, PSN-PC exhibited the more potent antimicrobial activity, especially against the Gram positive bacterium, *S. aureus*, and anti-yeast activity with the lowest MIC of 2 µM, making PSN-PC the most effective compound among the majority of reported phylloseptins [8,10,11,12,13,14,15]. Some studies have also evaluated the susceptibility of sessile *S. aureus* in biofilm, resulting in chronic and recurring bacterial infections in humans and high levels of antibiotic tolerance [28]. PSN-PC significantly eradicated the biofilm of *S. aureus* at 8 µM, showing a slightly weaker effectiveness compared to its bioactivity against planktonic *S. aureus*. PSN-PC is considered to remove microorganisms by interacting with bacterial membranes and causing pore formation, lysis and microbicidal death, which was consistent with the antimicrobial strategy [29].

Interestingly, because of the fact that cancer cells generally have more phosphatidylserine in the outer leaflet of the membrane than in normal cells, they are more susceptible to the lytic action of AMPs [30]. However, PSN-PC shows a broad-spectrum antimicrobial activity and no effect on tested cancer cells except for NCl-H157. The reason for selective killing of bacteria and cancer cells by PSN-PC could be related to distinct compositions of bacterial and cancer cell membranes and different structural transformations of peptides when in contact with different membranes. A possible explanation for this might be that the negative charges carried by NCI-H157 are more than that of other cancer cells tested. In fact, a recent study has shown that DRS-B2 resulted in the necrosis of PC3 cancer cells through a rapid membrane disruption [31]. The accepted antimicrobial mechanism is complicated and non-specific, not involving binding to a receptor [32]. The cationic compounds can interact with the negatively charged components of bacterial and cancer cells and the interaction may influence the strong link and selective disruption of bacterial and cancer cell membranes [33]. The non-specific interaction of AMPs with cell membranes results in the loss of membrane integrity, leakage of intercellular contents which eventually producing cell death. On the other hand, the cross-resistance of microorganisms to AMPs would be less likely to occur since the mechanisms of mode of amphipathic natural peptides are unrelated to conventional antibiotics, which is consistent with the finding of Stark et al. [34].

In conclusion, a novel peptide PSN-PC was first isolated from the skin secretion of *Phyllomedusa camba* and properly tested against various microbes. PSN-PC showed potent activity against bacteria, fungi and exhibited anti-tumor activity against the non-small cell lung cancer cell line NCl-H157. Furthermore, it demonstrated the significant capacity of eradicating *S. aureus* biofilm infections. In fact, the multifunctional peptide is the most effective AMP among most of the phylloseptins from natural sources. Its excellent broad-spectrum antimicrobial activities make it a possible candidate for antimicrobial and anti-biofilm agents. These findings may provide useful clues regarding new agents for treating recurrent infections.

## 4. Materials and Methods 

### 4.1. Acquisition of Skin Secretion

Specimens of the frog *Phyllomedusa camba* (*n* = 3) were obtained from commercial sources. The skin secretion was obtained via mild squeezing and massaging the dorsal skin of frogs, after which the frogs were released. The viscous white skin secretion was washed from the skin using deionised water, snap-frozen in liquid nitrogen, lyophilised and stored at −20 °C before analysis. The study was performed according to the guidelines in the UK Animal (Scientific Procedures) Act 1986, project license PPL 2694, issued by the Department of Health, Social Services and Public Safety, Northern Ireland. Procedures had been vetted by the IACUC of Queen’s University Belfast, and approved on 1 March 2011.

### 4.2. “Shotgun” Cloning of a Phyllomedusa camba Skin Secretion-Derived cDNA Library

Five milligrams sample of lyophilised *Phyllomedusa camba* skin secretion were dissolved in 1 mL of cell lysis/mRNA stabilisation buffer (Dynal, Merseyside, UK). Then the polyadenylated mRNA was isolated utilizing magnetic oligo-dT beads under the guidance of the manufacturer (Dynal, Merseyside, UK) and the isolated mRNA was subsequently subjected to 5’- and 3’-rapid amplification of cDNA end (RACE) procedures to acquire full-length prepropeptide nucleic acid sequence data by using a SMART-RACE kit (Clontech, Oxford, UK) essentially as outlined by the manufacturer. Briefly, the 3’-RACE reactions employed a nested universal (NUP) primer and degenerate sense primer (S1; 5’-ACTTTCYGAWTTRYAAGMCCAAABATG-3’ (Y = C/T, W = A/T, R = A/G, M = A/C, B = T/C/G) that were designed to highly-conserved segments of the signal peptides of cDNAs cloned previously from other *Phyllomedusa* frogs within our group [35]. The PCR cycling procedure included an initial denaturation at 94 °C for 90 s and 35 cycles for further denaturation at 94 °C lasting for 30 s, after which followed primer annealing for 30 s at 58 °C and extension for 180 s at 72 °C. PCR products were gel-purified and cloned using a pGEM-T vector system (Promega Corporation, Southampton, UK) and sequenced using an ABI 3100 automated sequencer (Applied Biosystems, Foster City, CA, USA).

### 4.3. Identification and Structural Analysis of PSN-PC

Five mg of lyophilised skin secretion of *Phyllomedusa camba*was dissolved in 0.5 mL of trifluoroacetic acid (TFA)/water and the supernatants were centrifuged at 2500× *g* for 5 min and pumped to an analytical reversed phase HPLC Jupiter C5 column (250 mm × 4.6 mm, Phenomenex, UK). All fractions were eluted from the column using a gradient programme which ran over 240 min at a flow rate of 1 mL/min from water/TFA (99.95/0.05, *v*/*v*) to acetonitrile/water/TFA (80/19.95/0.05; *v*/*v*/*v*). A Cecil CE4200 Adept gradient reverse phase HPLC (Cecil, Cambridge, UK) was used to collect fractions at 1 min intervals. All fractions were analysed by time-of-flight mass spectrometry (MALDI-TOF MS) (Voyager DE, Perspective Biosystems, Foster City, CA, USA) with _α-cyano-4-hydroxycinnamic acid (CHCA) as the matrix in positive mode. The instrument was calibrated by standards and set accuracy was ±0.1%. The peptide containing a molecular mass coincident with that predicted from cloned cDNA, was injected into a LCQ-Fleet electrospray ion-trap mass spectrometer to determine its primary structure by MS/MS fragmentation (Thermo Fisher Scientific, San Francisco, CA, USA).

### 4.4. Solid-Phase Peptide Synthesis

The replicate was synthesised by solid-phase Fmoc chemistry using Rink amide resin in a Tribute automated peptide synthesiser (Protein Technologies, Tucson, AZ, USA) when the unequivocal primary structure of the novel peptide had been confirmed. The reaction involved deprotection of the Fmoc groups from the amino acids and coupling of peptide bonds. When the synthesis cycles were completed, the peptide was cleaved from the resin using trifluoroacetic acid (TFA), triisopropylsilane (TIPS) and water (95/2.5/2.5, *v*/*v*/*v*) for 25 mL/g resin. The authenticity of the purified synthetic PSN-PC was verified by RP-HPLC with an analytical Jupiter C5 column (250 mm × 4.6 mm, Phenomenex, UK). The sample was eluted with a linear gradient from water/TFA (99.95/0.05, *v*/*v*) to acetonitrile/water/TFA (80/19.95/0.05; *v*/*v*/*v*) in 40 min at a flow rate of 1 mL/min. The degree of purity and authentication of the purified synthetic peptide was determined by RP-HPLC (Cecil, Cambridge, UK) and MALDI-TOF MS (Voyager DE, Perspective Biosystems, Foster City, CA, USA) as previously described [36].

### 4.5. Circular Dichroism (CD) Spectroscopy

The secondary structure of the peptide was determined using a JASCO J-815 CD spectrometer (Jasco, Essex, UK). It was dissolved in 10 mM ammonium acetate and 10 mM ammonium acetate with 50% trifluoroethanol (TFE), respectively and then was prepared at 100 µM in a 1 mm high precision quartz cell (Hellma Analytics, Essex, UK). CD spectra were recorded at a wavelength ranging from 190 nm to 250 nm with a 100 nm/min scan speed. The parameters were set as 1 nm bandwidth and 0.5 nm data pitch. 

We supplemented the secondary structure prediction with some bioinformatics tools. A vividly visual plot containing some further details about alpha helices was demonstrated by helical wheel projections. The significant properties of the novel peptide were predicted by Heliquest (http://heliquest.ipmc.cnrs.fr/cgi-bin/ComputParams.py). Additionally, the I-TASSER webserver [37,38] was utilized to simulate a 3D model. Overall qualities of the predicted model were evaluated by z-scores using ProSA [39,40].

### 4.6. Antimicrobial Activities 

The broth dilution method [41] was referred and slightly modified to evaluate the antimicrobial activity of the novel peptide. In short, different concentrations of the peptide ranging from 1 to 512 µM were incubated with microorganisms under defined conditions. These microorganisms consisted of the Gram-positive bacteria *Staphylococcus aureus* (*S. aureus*) (NCTC10788), methicillin-resistant *Staphylococcus aureus* (MRSA) (NCTC 12493); the Gram-negative bacteria *Escherichia coli* (*E. coli*) (NCTC 10418), *Pseudomonas aeruginosa* (*P. aeruginosa*) (ATCC 27853) and the yeast *Candida albicans* (*C. albicans*) (NCYC 1467). The microbial suspension which had been inoculated and cultured overnight was diluted with fresh Mueller-Hinton broth (MHB) to a concentration of 1 × 10^6^ colony-forming units (CFU)/mL. The sample was initially dissolved in a stock solution of 1024 µM in sodium phosphate-buffered saline (PBS, pH 7.2) and subsequently double-diluted in MHB to achieve final concentrations of the peptide from 512 to 1 µM. Peptide solutions were incubated with growth cultures mentioned above (10^6^ CFU/mL) in 96-well plates for 18 h at 37 °C. After that, the absorbance values of the wells of the 96-well plates were determined at 550 nm using a Synergy HT plate reader (Biolise BioTek, Winooski, VT, USA) and the MIC was defined as the lowest concentration of peptide that resulted in no apparent growth of the microorganism. From these wells, 10 µL of the overnight culture was added to a Mueller-Hinton agar (MHA) plate and cultured at 37 °C for 16–20 h. The lowest concentrations that showed no evidence of colony growth were considered as the MBCs. 

### 4.7. Anti-Biofilm Assays with S. aureus

MBEC assays were based on a modified 2,3,5-triphenyl tetrazolium chloride (TTC) method [42]. Overnight cultures were washed with sterile PBS and diluted with fresh broth to 10^6^ CFU/mL. For the MBEC assay, 200 µL of inoculum was placed in a flat-bottomed microtiter plate for 48 h to form mature biofilms. Following sufficient growth time, mature biofilms were washed twice to remove the planktonic cells and incubated with a series of peptide concentrations (1–512 µM) at 37 °C for 20–24 h. After sufficient growth, plates were washed twice with sterile PBS followed by the addition of fresh medium (200 µL per well) and stained with 50 µL 1% TTC (g/v) solution for 5 h. After incubation, 200 µL of the supernatant from each well was transferred to a new plate, and their absorbance values were measured at 470 nm using a Synergy HT plate reader (Biolise BioTek, Winooski, VT, USA).

### 4.8. Time-Killing Assay with S. aureus

Time-killing curve analyses were carried out by culturing *S. aureus* in MHB medium in the presence of three antimicrobial concentrations in doubling dilutions ranging from 4 × MIC to 1 × MIC. The MIC value referred to the results of antimicrobial assay mentioned above. The sample was initially dissolved and double-diluted in MHB to achieve final concentrations of the peptide from 8 µM to 2 µM. Bacterial cells which were inoculated and cultured overnight were diluted with peptide-treated MHB to a concentration of 1 × 10^6^ CFU/mL. Growing bacteria were removed at specified time points and diluted in sterile PBS in six subsequent 1:10 dilutions (20 μL culture in 180 μL diluent). Twenty μL droplets of each dilution were spotted on MHA plate and cultured at 37 °C. For every concentration and time point, colonies were counted for the first dilution that resulted in a countable range of 3–100 colonies and the CFU/mL was calculated.

### 4.9. Haemolytic Assay

–A 96 well plate reader (Biolise BioTek EL808) was used for measuring the optical density of lysis of cells at 550 nm. A 2% suspension (*v*/*v*) was formed with prewashed defibrinated horse erythrocytes (TCS Biosciences Ltd., Botolph Claydon, Buckingham, UK) and sodium phosphate-buffered saline (PBS). Peptides were incubated with this 2% suspension in a final concentration range from 1 to 512 µM, and all the tested samples were kept at a constant 37 °C for 2 h. Negative controls employed were PBS and a 2% red cell suspension in equal volume while positive controls were PBS containing 2% of the non-ionic detergent, Triton X-100 (Sigma-Aldrich, St. Louis, MO, USA) together with a 2% red cell suspension. The sample supernatants were used to assess the extent of haemolysis by measuring the optical density (OD) value at 550 nm.

The haemolysis ratio was calculated using the following equation: % haemolysis = (A − A_0_) / (A_X_ − A0) × 100, where ‘A’ is absorbance with peptides of different concentrations, ‘A0’ is absorbance with negative controls and ‘AX’ is absorbance with positive controls. All OD values were measured at 550 nm.

### 4.10. Bacterial Cell Membrane Permeability Assay of PSN-PC Using S. aureus

The membrane permeability assay was carried out using SYTOX Green Nucleic Acid Stain (Life technologies, Carlsbad, CA, USA) [43]. Bacteria were incubated in Tryptic Soy Broth (TSB) (Sigma-Aldrich, St. Louis, MO, USA) at 37 °C overnight, after which 200 µL of bacterial culture was inoculated into 25 mL TSB and incubated at 37 °C for 2.5 h to achieve the logarithmic growth phase. Next, the supernatant was removed by centrifugation at 1000× *g* for 10 min at 4 °C, and bacterial cells were washed twice with 5% TSB in 0.85% NaCl solution. The washed bacterial cells were suspended in 5%TSB to achieve 1 × 10^8^ CFU/mL which was 0.7 at OD 590 nm. Each well of the sample groups in a black 96 well plate (Fisher Scientific, Leicestershire, UK) contained a volume of 50 µL of bacterial suspension and 50 µL of peptide solution. Each well of the negative control group was constituted by a volume of 50 µL of bacterial suspension and 40 µL of 5% TSB. The positive control group was made by a volume of 50 µL of permeabilised bacterial cell suspension by using 70% isopropanol and 40 µL of 5% TSB. 10 µL of SYTOX green nucleic acid stain was added to each well to form a final concentration of 5 µM. Meanwhile, the background fluorescence was measured using a volume of 90 µL 5% TSB and 10 µL SYTOX green nucleic acid stain at the same concentration. The black plate was incubated for 2 h at 37 °C in the dark. The fluorescent intensity of each well was recorded using an ELISA plate reader (Biolise BioTek EL808, Winooski, VT, USA) with excitation at 485 nm and emission at 528 nm.

### 4.11. Cells Lines and Cell Culture

The human breast cancer cell lines MB435s, MCF-7, the human prostate cancer cell Line PC3, the non-small cell lung cancer cell line NCl-H157 and the human neuropongioma cell line U251MG, were separately cultured employing RPMI-1640 culture medium (Invitrogen, Paisley, UK), or Dulbecco’s Modified Eagle’s Medium (DMEM) (Sigma, St. Louis, MO, USA), with 1% penicillin streptomycin solution (Sigma) and 10% fetal bovine serum (FBS) (Sigma) added. The human microvessel endothelial cell HMEC-1 was employed to evaluate the cytotoxicity of the peptide against normal human cells. These cells were grown in 10% FBS, 10 ng/mL EGF, 10 mM l-Glutamine, 1% penicillin streptomycin supplemented MCDB131 medium (Gibco, Paisley, UK). The selected cells were inoculated into 90 mm culture dishes (Nunc, Roskilde, Denmark) or 75 cm^2^ culture flasks (Nunc). Following this, flasks were placed in an incubator with a humidified environment containing 5% CO_2_.

### 4.12. Assessment of Cancer Cell Anti-Proliferative Activity Using the MTT Cell Viability Assay

Cancer cell line proliferation and viability were assessed using the MTT cell viability assay [44]. Briefly, each of the cancer cell lines was seeded at a density of 5 × 10^3^ cells per well onto 96 well plates. Following this, cell lines were prepared with gradient concentrations of peptide and incubated for 24 h. After this, 10 µL of 5 mg/mL yellow coloured MTT solution (Sigma) were added to all wells and incubated again for 4 h. Once the supernatants were removed by a syringe, 100 µL of DMSO were added to all wells after gently agitating to completely mix the formazan crystals that had developed. A Synergy HT plate reader (BioTek, Winooski, VT, USA) was set at 570 nm for recording the absorbance.

### 4.13. Statistical Analysis

Data were subjected to statistical analysis using Prism (Version 5.0; GraphPad Software Inc., San Diego, CA, USA). Error bars in the graphs represent standard error of the mean (SEM) with experiments performed on more than three sets of replicates.

## Figures and Tables

**Table 1:** Minimum inhibitory concentrations (MICs) and minimum bactericidal concentrations (MBCs) of PSN-PC.

Strains	*S. aureus*	MRSA	*C. albicans*	*E. coli*	*P. aeruginosa*
MIC (µM)	2	2	2	8	32
MBC (µM)	4	4	2	8	64

**Table 2:** Minimum inhibitory concentrations (MICs) and corresponding physicochemical parameters of natural phylloseptin AMPs against specified microorganisms.

Peptide Name	MIC (mg.L^−^^1^/µM)			<H>	<µH>	Net Charge
	*S. aureus*	*E. coli*	*C. albicans*			
Phylloseptin-PHa	64/33	>512/>264	256/131.9	0.799	0.457	0
PS-PT	55/26.4	55/26.4	55/26.4	0.686	0.438	+ 2
PLS-S2	12.7/6.3	50.9/25	ND	0.801	0.548	+ 1
PLS-S4	12.5/6.3	50.1/25	ND	0.789	0.519	+ 1
Phylloseptin-7	12/6	12/6	ND	0.745	0.513	+ 1
Phylloseptin-PBa	8/4.2	128/67.6	8/4.2	0.711	0.637	+ 2
Phylloseptin-PTa	8/4.1	32/16.6	4/2.1	0.740	0.577	+ 2
PS-Co	8/4.1	128/64.9	16/8.1	0.706	0.636	+ 2
PS-Du	8/3.90	128/62.5	16/7.8	0.725	0.624	+ 2
PSN-PC	4/2	16/8	4/2	0.754	0.563	+ 2

<H>: Hydrophobicity; <μH>: Hydrophobic moment; ND: not detected. MICs came from publications [8,10,11,12,13,14,15], and their physicochemical parameters were calculated by online analysis tool, HeliQuest.

## Abbreviations

AMPs	antimicrobial peptide
CD	circular dichroism
DMEM	Dulbecco’s modified eagle’s medium
FBS	fetal bovine serum
MALDI-TOF MS	matrix-assisted laser desorption/ionization time of flight mass spectrometry
MBC	minimum bactericidal concentration
MBEC	minimal biofilm eradication concentration
MHA	Mueller-Hinton agar
MHB	Mueller-Hinton broth
MIC	minimum inhibitory concentration
MS/MS	tandem mass spectrometry
NUP	nested universal primer
PBS	phosphate-buffered saline
RP-HPLC	reverse-phase high performance liquid chromatography
TFA	trifluoroacetic acid
TFE	trifluoroethanol
TIPS	triisopropylsilane
TSB	tryptic soy broth
TTC	2,3,5-triphenyl tetrazolium chloride
